# Supplementary material for: Complexome profiling of the Chlamydomonas psb28 mutant reveals TEF5 as an early PSII assembly factor
Source: Plant Cell. 2025 Mar 17;37(6):koaf055. doi: 10.1093/plcell/koaf055 (PMC12164590; doi:10.1093/plcell/koaf055)
Supplement: koaf055_Supplementary_Data [file koaf055_supplementary_data.zip › psb28_interactive.zip!/plots/intensity/qualityPlots/peptides/REC8_Cre12.g554650.t1.2.html]

### Cre12.g554650.t1.2

Trivial name: REC8  
SeqCoverage [median]: 1.50  
Mass: 116.28 kDa  
  
 

| Sequence | MissedCleavage | Mass [Da] | wtIntensity | muIntensity | Score |
| --- | --- | --- | --- | --- | --- |
| TAAGASAGAAGTSAGGR | 0 | 1332.64 | 54476.65 | 63650.97 | 84.31 |
|  |  |  | 54476.7 | 63651.0 |  |
